# Reduced representative methylome profiling of cell-free DNA for breast cancer detection

**DOI:** 10.1186/s13148-024-01641-x

**Published:** 2024-02-27

**Authors:** Qingmo Yang, Xingqiang Zhu, Yulu Liu, Zhi He, Huan Xu, Hailing Zheng, Zhiming Huang, Dan Wang, Xiaofang Lin, Ping Guo, Hongliang Chen

**Affiliations:** 1 The First Affiliated Hospital of Xiamen University, School of Medicine, Xiamen University, Xiamen, Fujian China; 2 Xiamen Vangenes Biotechnology Co., Ltd, Xiamen, 361015 Fujian China; 3 School of Life Sciences, Xiamen University, Xiamen, 361102 Fujian China; 4 Xiamen Huazao Biotechnology Co., Ltd, Xiamen, 361015 Fujian China

## Abstract

**Background:**

Whole-genome methylation sequencing of cfDNA is not cost-effective for tumor detection. Here, we introduce reduced representative methylome profiling (RRMP), which employs restriction enzyme for depletion of AT-rich sequence to achieve enrichment and deep sequencing of CG-rich sequences.

**Methods:**

We first verified the ability of RRMP to enrich CG-rich sequences using tumor cell genomic DNA and analyzed differential methylation regions between tumor cells and normal whole blood cells. We then analyzed cfDNA from 29 breast cancer patients and 27 non-breast cancer individuals to detect breast cancer by building machine learning models.

**Results:**

RRMP captured 81.9% CpG islands and 75.2% gene promoters when sequenced to 10 billion base pairs, with an enrichment efficiency being comparable to RRBS. RRMP allowed us to assess DNA methylation changes between tumor cells and whole blood cells. Applying our approach to cfDNA from 29 breast cancer patients and 27 non-breast cancer individuals, we developed machine learning models that could discriminate between breast cancer and non-breast cancer controls (AUC = 0.85), suggesting possibilities for truly non-invasive cancer detection.

**Conclusions:**

We developed a new method to achieve reduced representative methylome profiling of cell-free DNA for tumor detection.

**Supplementary Information:**

The online version contains supplementary material available at 10.1186/s13148-024-01641-x.

## Introduction

Cell-free DNA (cfDNA) in plasma is primarily derived from the apoptosis or necrosis of cells in a variety of tissues throughout the body [1, 2]. Circulating tumor DNA (ctDNA) from tumor cells is used as a biomarker for liquid biopsy testing approaches for tumor detection [3–5]. Several studies have identified specific ctDNA features that may be useful for tumor detection by cfDNA profiling, such as mutations, DNA methylation, fragmentation, copy number aberrations, and end motifs [6–8]. In particular, methylation modifications in ctDNA are widely used as biomarkers for tumor detection [9, 10].

The discovery of ctDNA markers provides an opportunity for tumor liquid biopsy, but there are still many challenges. First, the low level of ctDNA in plasma, 0.1% or even less in the early stages of tumor development, is a technical challenge for early cancer detection [11]. Second, tumors of different types, subtypes, or etiologies have different patterns of methylation aberration. This provides an opportunity for tumor-of-origin localization, but also poses a challenge for marker selection. An increase in the depth of coverage and number of methylation markers may improve the sensitivity of ctDNA detection and the accuracy of tumor-of-origin localization [12]. Targeted capture allows deep sequencing of the custom target region, but only a portion of the ctDNA can be analyzed, which may lead to false negative results.

The discovery or selection of methylation markers requires comprehensive profiling DNA methylation. However, CpG sparse regions out of CpG islands consume most sequencing capacity of whole-genome bisulfite sequencing (WGBS) [13, 14]. Reduced representative bisulfite sequencing (RRBS) enriches CpG-dense regions by restriction digest, requiring reduced sequencing throughput [15]. But RRBS needs relatively large amounts of genomic DNA input and is not suitable for analyzing low amounts of highly fragmented cfDNA [16]. Extended-representation bisulfite sequencing (XRBS) expands coverage over gene regulatory elements and is compatible with low-amount genomic DNA samples, but it has not been used to analyze cfDNA [17]. Here, we present a versatile strategy for reduced representative methylome profiling (RRMP), which is compatible with both genomic DNA and highly fragmented cfDNA.

## Materials and methods

### Cells

K562 (RRID:CVCL_0004), Calu-1 (CVCL_0608), NCI-H460 (CVCL_0459), Hep 3B2.1-7 (RRID:CVCL_0326), Li-7 (CVCL_3840), Caco-2 (CVCL_0025), HT29 (CVCL_A8EZ), BT474 (CVCL_0179), BT-549 (CVCL_1092) and HGC-27 (CVCL_1279) were obtained from NICR (The Chinese National Infrastructure of Cell Line Resource). Cell line authentication was performed using STR profiling kit (HUMDNATYPING SYSTEM, BGI). Genomic DNA was purified with DNeasy Blood & Tissue Kit (Qiagen).

### Human participants and sample acquisition

Plasma samples were collected from female patients with BC or benign disease before any treatment at the First Affiliated Hospital, Xiamen University, between December 2021 and December 2022. The present study was performed under the Helsinki Declaration and was approved by the Ethics Committee of the First Affiliated Hospital, Xiamen University (reference number: XMYY-2021KYSB209). Informed consent was obtained from all participants or their families.

### Blood sample processing and cfDNA purification

cfDNA samples were processed by the following method. Peripheral blood was collected from all volunteers using cfDNA Blood Collection Tube (Zhixuan Biotech). Plasma was separated by centrifugation at 1600 g for 10 min and transferred to microcentrifuge tubes. After then, centrifugation was done at 16,000 g for 10 min to remove cellular debris. The supernatant was divided into 2-ml aliquots and stored at − 80 °C until the time of DNA extraction. cfDNA was extracted from 2-ml plasma for each participant using Plasma Cell-Free DNA Extraction Kit (Concert). cfDNA concentration was measured using Qubit dsDNA High Sensitivity Assay Kit (Thermo Fisher).

### RRMP library preparation

**Fig. 1:**
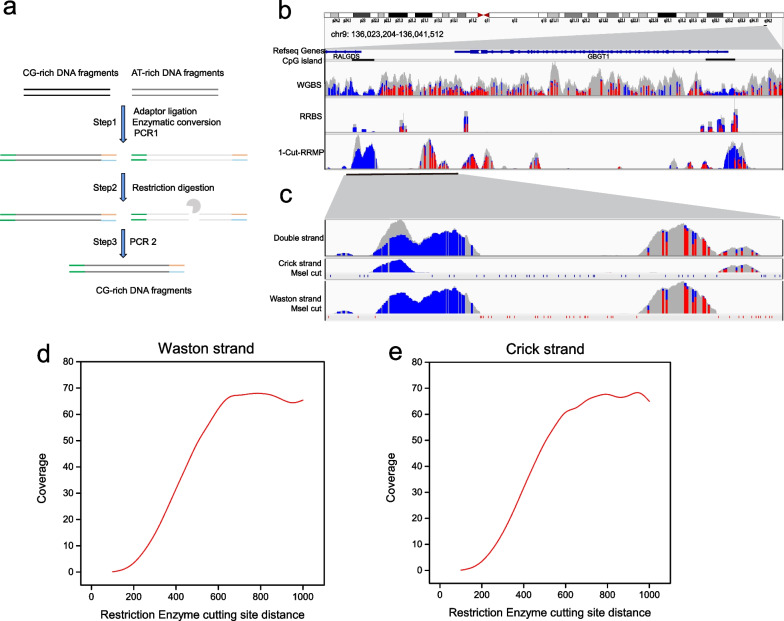
A reduced representative methylome profiling method. **a** Schematic representation of RRMP. **b** Genome plot for the GBGT1 gene locus compares read coverage between 1-cut RRMP (K562) and public WGBS (K562) and RRBS (SW1353) datasets. Boxes represent reads, and unmethylated (blue) and methylated (red) CpGs are indicated. CpG islands are indicated. **c** Genome plot for the GBGT1 gene locus shows distribution of 1-cut RRMP reads and MseI targeted sites in both Watson and Crick strand. **d**, **e** Plot shows the average coverage depth as a function of distance between the upstream and downstream enzyme recognition sites in both Watson (**d**) and Crick (**e**) strand

The process for RRMP library preparation is shown in Fig. 1a. First, EM-seq library was prepared as described previously [18]. In brief, mechanically fragmented cell genomic DNA or cfDNA was treated with VAHTS Universal DNA library Prep Kit (New England BioLabs) for end-repair, A-tailing, and ligation of EM-seq adaptor (New England BioLabs). The ligated sample was methyl-converted with EM-seq Conversion Module (New England BioLabs) per the manufacturer’s protocol. Methyl-converted DNA was purified and amplified using NEBNext Unique Dual Index Primers (New England BioLabs) and KAPA HiFi HotStart Uracil + ReadyMix (KAPA biosystems). No more than 15 EM-seq libraries (150 ng per sample) were pooled together and subjected to restriction digest with MseI or a combination of MluCI, MseI, SspI, PsiI with incubation time of more than 30 min. Following purification, DNA products were amplified for three cycles using KAPA HiFi HotStart Uracil + ReadyMix (KAPA biosystems). To achieve complete digestion, the digestion and amplification were repeated twice.

### Next-generation sequencing and data processing

All RRMP and EM-seq libraries were sequenced using the Illumina Novaseq 6000 platform by Mingma Technologies Inc (Shanghai). Paired-end data were generated using 2 × 150 sequencing cycles, as detailed in Additional file 1: Table S1. The raw sequencing FASTQ files were processed using *Fastp* (0.23.2) to trim Illumina-specific adapters with default parameters and low-quality sequences with parameters of -u 20 and -q 20 [19]. Mapping to the human reference genome hg19 of the processed sequencing reads was performed using the *bismark* (0.23.0) [20]. Incomplete converted reads with more than 3 CHs were removed using *filter_non_conversion* (*bismark*). Average DNA methylation values and coverage within CpG islands, promoters (1 kb upstream and 500 bp downstream of all transcription start sites of protein-coding genes), and genomic 100-kb windows were used for many analyses.

### Differentially methylated regions analysis

RRMP libraries from 9 cancer cell lines and 3 whole blood cell (WBC) samples from healthy donors were used to analyze differentially methylated regions (DMRs) using *dmrfinder* [21]*.* Genomic regions (≤ 300 bp) detected at least 10 × coverage in all datasets were retained for further analysis. Average methylation beta value was calculated for each type of samples and used for DMR analysis. We obtained DMRs at *p* < 0.0000001, Δ beta value > 0.5 for each type of tumor cell lines compared to WBC samples; DMRs at *p* < 0.05, Δ beta value > 0.2 for all types of tumor cell lines compared to WBC samples; and DMRs at *p* < 0.05, Δ beta value > 0.33 for each type of tumor cell lines compared to WBC samples and other type of tumor cell lines.

RRMP libraries of NCI-H460, HEP3B, HT29, and BT549 were uses to parse differentially methylated promoters. *BEDTools intersect* and *BEDTools groupby* were used to analyze the methylation values of the upstream and downstream 4 kb regions of TSS and then used *methylkit* to obtain the methylation regions specific to each cell (*q* value < 0.01, Δ beta > 0.5) [22, 23].

### Correlation analysis of cell-specific DNA methylation with histone modification

H3K4me3, H3K27ac ChIP-Seq datasets and control datasets for HT29 and NCI-H460 were downloaded from SRA (Additional file 1: Table S2). *macs2 callpeak* was used to obtain H3K4me3 and H3K27ac peak files of two cells from alignments results to human genome (*q* value < 0.01) [24]. Methylation levels of 4 kb regions upstream and downstream of these peaks were calculated using RRMP data and the *methylkit* tool to pick regions with differential methylation (*q* value < 0.01, Δ beta > 0.5). The number of DMRs with H3K4me3 and H3K27ac peaks in each cell was used to compute Fisher exact test P values to determine correlation.

### Development of BC detection model

Autosomal CpGs covering more than 100 × in more than 80% of the samples were included for further analysis. Samples with more than 20% of these sites covered less than 100 × were excluded. CpGs in the 2 kb upstream and downstream of TSS with variance ≥ 0.009 were included for feature selection and construction of breast cancer detection model using a supervised machine learning approach with leave-one-out (LOO) cross-validation [25, 26]. The logistic regression output for each validated sample was the prediction tumor score.

## Results

### A versatile method for reduced representative methylome profiling

Bisulfite treatment of DNA converts unmethylated C into T without changing 5mC and 5hmC, resulting in a decrease in the CG content. We reasoned that it is possible to enrich CpG dense regions by eliminating AT-rich fragments following C to T conversion. In silico analysis of a WGBS data from SRA showed that the removal of AT-rich reads could significantly increase the CG content of the remaining reads and the proportions of the remaining reads mapping to CpG islands and promoters, the enrichment effect of which was comparable to that of RRBS (Additional file 2: Fig. S1). Therefore, we designed RRMP method by using DNA endonucleases targeting AT-rich sequences to treat C-to-T converted library, which is compatible with both genomic DNA and highly fragmented cfDNA (Fig. 1a).

We prepared an EM-seq library from 30 ng of fragmented genomic DNA from K562 cells [27]. The DNA library was digested using a single enzyme (MseI) and a combination of four enzymes (MseI, MluCI, SspI, and PsiI) to generate 1-cut and 4-cut RRMP libraries, respectively. Approximately 75 million reads were sequenced for each RRMP library (Additional file 1: Table S1). Figure 1b and Additional file 2: Figure S2 show representative regions of the RRMP datasets compared to published data. Similar to the RRBS, the reads of the RRMP datasets were enriched in the CpG island regions, with depletion of reads at the vicinity of cut sites (Fig. 1c). The coverage depth of genomic regions with sparse cut sites is higher than those with dense cut sites, indicating efficient genome-wide elimination of AT-rich reads (Fig. 1d, e).

### RRMP enhanced coverage over CpG islands and promoters

**Fig. 2:**
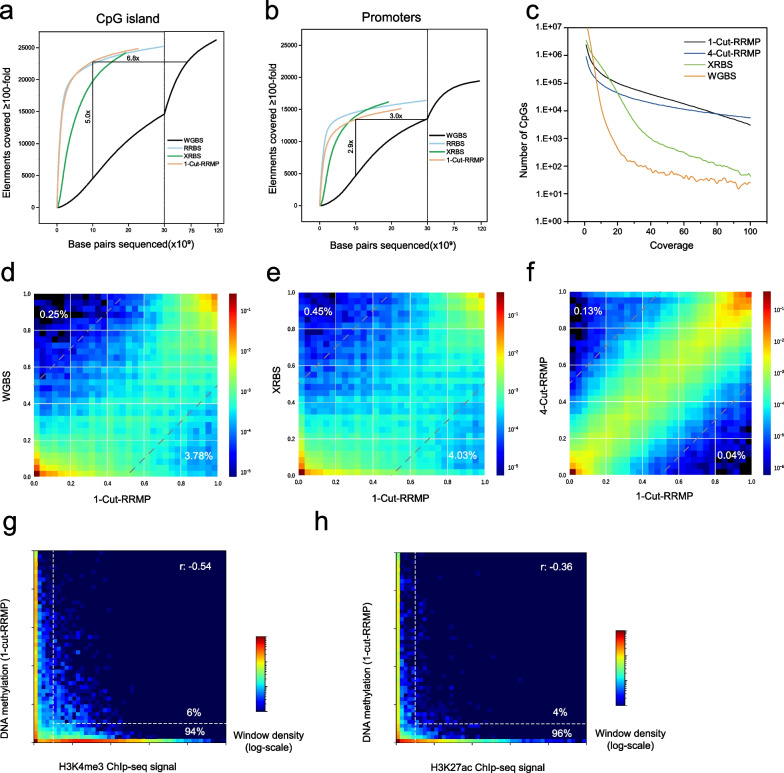
RRMP analysis of methylation in CpG island and promoters. **a**,** b** Plots show the number of CpG islands (**a**) or promoters (**b**) with at least 100-fold combined coverage as a function of sequencing depth (x axis) for 1-cut RRMP (K562), XRBS (K562), WGBS (K562), and RRBS (SW1353). Enrichment for functional elements at a uniform sequencing depth of 10 billion base pairs is indicated. Vertical gray line indicates break in x-axis scale. **c**, Plot shows coverage depth of CpGs in WGBS, XRBS, 1-cut RRMP, and 4-cut RRMP at a uniform sequencing depth of 10 billion base pairs. **d**–**f** Heat maps compare individual CpG methylation values between 1-cut RRMP and WGBS (d, *r* = 0.88), 1 cut-RRMP and XRBS (e, *r* = 0.85), 1 cut-RRMP and 4-cut RRMP (f, *r* = 0.98) for K562 cells. Analysis limited to CpGs with at least 20-fold coverage (*n* = 128,501, 160,804 and 1,659,714 CpGs). Percentages indicate the fraction of CpGs that differed between conditions (difference in beta values > 0.5). **g**, **h** Heatmaps compare average DNA methylation values from 1-cut RRMP datasets with signal for H3K4me3 (**g**) and H3K27ac (**h**) for K562 cells. Percentages indicate the fraction of hypermethylated (beta value >  = 10%) and hypomethylated(beta value < 10%) regions with Chip-seq signal > 1

We further evaluated RRMP coverage in CpG islands and promoter regions, in comparison with WGBS, RRBS, and XRBS. Coverage was normalized for each method via downsampling. An element of CpG island or promoter was considered to be covered if CpGs in this element accumulated to ≥ 100-fold coverage [17]. With these criteria, RRMP captured more CpG islands and promoters than WGBS at lower sequencing throughput. When downsampled to 10 billion base pairs of sequencing data, 1-cut RRMP captured 22,797 CpG islands and 13,442 promoters, 5.0 and 2.9 times the coverage of WGBS **(**Fig. 2a, b). RRMP showed similar enrichment efficiency to RRBS and XRBS. When sequenced to saturation (~ 23 billion base pairs), 1cut-RRMP captured 81.9% CpG islands and 75.2% gene promoters (compared to 83.1% and 81.7% for RRBS, Fig. 2a, b). Similarly, 4-cut RRMP captured about 19,332 CpG islands and 10,810 promoters at sequencing data of 10 billion base pairs, 4.3 and 2.3 times the coverage of WGBS (Additional file 2: Fig S3 a-b). At lower sequencing throughput, RRMP captured more CpGs in the CpG islands and promoters with higher coverage depth, comparing to WGBS (Fig. 2c and Additional file 2: Fig S3 c-e).

High concordance in DNA methylation values of individual CpGs was confirmed between two RRMP datasets and between 1cut RRMP and two public datasets of K562 cells (Fig. 2d–e). RRMP detected global hypomethylation in K562 cells, consistent with previous study (Additional file 2: Fig S3f) [17]. We overlaid RRMP data and published data for histone modifications of K562 cells. As expected, DNA methylation was negatively correlated with histone H3K4me3 (*r* = − 0.54), which marks active and poised promoters and histone H3K27Ac mark (*r* = − 0.36), which is deposited at active enhancers (Fig. 2g, h) [28–30]. Most H3K4me3 (94%) and H3K27Ac (96%) with signal > 1 are found at regions with DNA hypomethylation (beta value < 10%).

### RRMP detects tumor cell specific DNA methylation

**Fig. 3:**
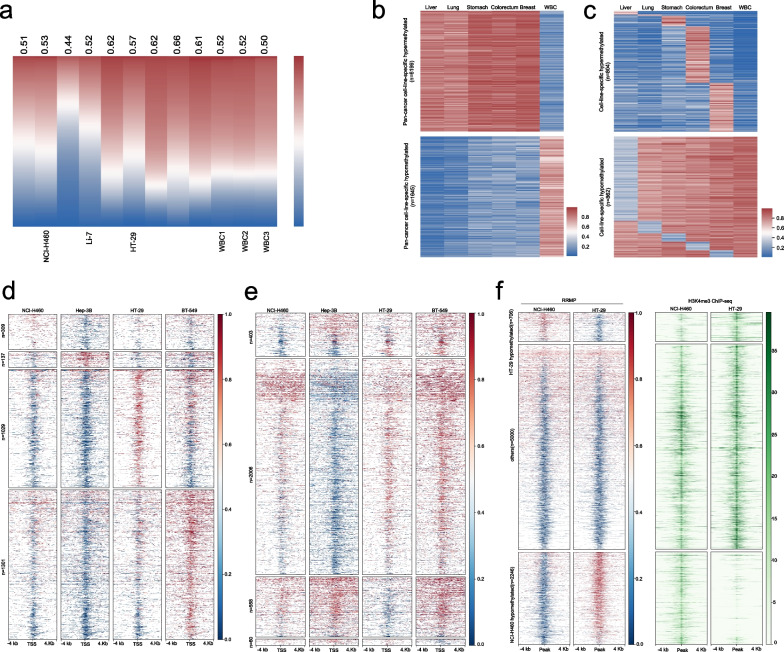
Analysis of tumor-related DNA methylation using RRMP. **a** Heat map shows genome-wide DNA methylation in 100-kb windows across 9 tumor cell lines and 3 WBC samples. Windows are sorted by decreasing DNA methylation for each cell line. Average methylation value for each sample is indicated below. **b** Heat map depicts hypermethylated and hypomethylated regions in tumor cells compared to WBC samples. **c** Heat map depicts hypermethylated and hypomethylated regions in each type of tumor cells compared to WBC samples and other tumor cell lines. **d, e** Heat map depicts 8-kb genomic regions (rows, *n* = 3972 promoters) centered at transcription start sites and divided into 100 equally sized bins. Panels show average methylation from 1000-cell XRBS profiles for the indicated cell types. Promoters (rows, ≥ 25-fold combined coverage in every cell line) are grouped by the cell line in which they are specifically hypermethylated (**d**) or hypomethylated (**e**). Hypomethylated promoters specific to K562 cells are downsampled for visualization. A full list of differentially methylated promoters is provided in Additional file 1: Table S3. **f** Heat map depicts 8-kb regions (rows, *n* = 15,202 regions) centered on H3K4me3 peaks identified in NCI-H460 and HT29 ChIP-seq datasets. Rows are ordered by DNA methylation difference between both cell lines. Panels show average methylation from 4-cut RRMP profiles and H3K4me3 signals for NCI-H460 and HT29. Cell-line-specific DNA hypomethylation correlates with H3K4me3 signal. Peaks not specifically hypomethylated in either cell line (‘Others’) were downsampled for visualization

We next used RRMP to compare methylation patterns across tumor cell lines. We generated 4-cut RRMP libraries using cell lines from lung cancer, liver cancer, colorectal cancer, breast cancer, gastric cancer, and three WBC samples from healthy donors and got average 50 million paired-end reads per library (Additional file 1: Table S1). RRMP detected global hypomethylation in Hep-3B cells (average beta value = 0.42) and global hypermethylation in other samples (average beta value = 0.50–0.62, Fig. 3a). The global methylation levels varied significantly among tumor cells (*r* = 0.39–0.77), while these were highly consistent among three whole blood samples (*r* = 0.92–0.93, Additional file 2: Fig S4a).

We next used RRMP data to analyze DMRs comparing tumor cells and whole blood cells (Additional file 2: Fig S4b and Additional file 1: Table S3-S7). We identified 6199 specific hypermethylated regions and 1645 hypomethylated regions in tumor cell lines compared to whole blood cells, which are expected to be biomarkers for the detection of multiple cancer types (Fig. 3b and Additional file 1: Table S8-S9). We also analyzed tumor-specific DMR for potential markers of tissue origin (Fig. 3c and Additional file 1: Table S10-S11). RRMP data analysis of four tumor cells yielded 2776 cell-specific hypermethylated promoters and 3027 cell-specific hypomethylated promoters (Fig. 3d, e).

Next, we investigated whether cell-specific methylation measured by RRMP could predict the status of histone modification in different cell lines. We aggregated 34,033 regions with H3K4me3 peak from HT29 and NCI-H460 cells covered in the RRMP data set. Of these peaks, 2.1% were specifically hypomethylated in HT29, and 6.6% were specifically hypomethylated in NCI-H460 cells, whereas the remaining 91.3% (others) were predominantly hypomethylated in both cell lines (Fig. 3f). Of regions specifically hypomethylated in HT29, 92.6% contained the H3K4me3 peak in HT29 cell, compared to 54.4% in NCI-H460 (*p* < 0.0001, Fisher's exact test; Fig. 3f). Of regions specifically hypomethylated in NCI-H460, 97.8% contained the H3K4me3 peak in NCI-H460, compared to 23.8% in HT29 (*p* < 0.0001; Fig. 3f). Hence, hypomethylation was associated with H3K4me3, suggesting that RRMP can be used to analyze the cell specific histone modifications. RRMP data were also used to analyze the negative correlation between methylation signals and enhancer signals, although RRMP coverage at the H3K27ac peaks was low (Additional file 2: Fig S5).

### Breast cancer detection using RRMP of cfDNA

**Fig. 4:**
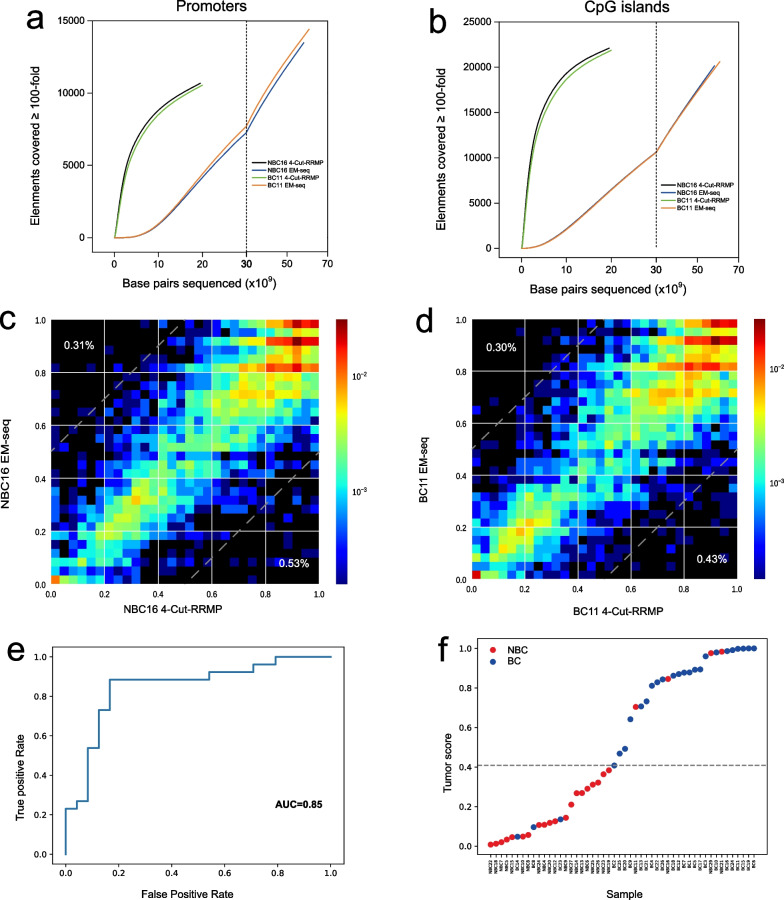
Breast cancer detection using RRMP analysis of cfDNA. **a**, **b** Plots show the number of CpG islands (**a**) or promoters (**b**) with at least 100-fold combined coverage as a function of sequencing depth (x axis) for 4-cut RRMP and EM-seq from two cfDNA samples NBC16 and BC11. Vertical gray line indicates break in x-axis scale. **c**, **d** Heat maps compare individual CpG methylation values between 4-cut RRMP and EM-seq for cfDNA samples NBC16 (**c**, *r* = 0.86) and BC11 (**d**, *r* = 0.87). Analysis limited to CpGs with at least tenfold coverage (*n* = 6358 and 7028 CpGs). Percentages indicate the fraction of CpGs that differed between conditions (difference in beta values > 0.5). **e** Receiver operator characteristic curves (ROC) and area under the curve (AUC) values. **f** LOO cancer prediction scores for BC and NBC. Dashed line represents probability score threshold. Samples with a probability score above this threshold were predicted as BC

We further investigated whether RRMP could be used to analyze cfDNA sample. We performed RRMP analysis of plasma samples from breast cancer patients (BC, *n* = 29) and non-breast cancer individuals (NBC, *n* = 27, Additional file 1: Table S1 and S12). The libraries were sequenced by an average of 75 million reads. To evaluate the enrichment effect of RRMP on cfDNA samples, we also sequenced the EM-seq library of two cfDNA samples (NBC16 and BC11), generating 194 and 203 million reads, respectively. Here, the library derived from one sample can be used for both genome-wide methylation sequencing and reduced representative methylome profiling, suggesting that RRMP facilitates sample re-use for multiple analyses. Compared to EM-seq datasets, RRMP highly enriched CpG island and promoter sequences and efficiently captured CpGs in CpG islands and promoters with higher coverage (Fig. 4a-b and Additional file 2: Fig S6 a-d). The methylation analysis was highly consistent between RRMP and EM-seq (Fig. 4c, d). Depletion of AT-rich regions by restriction enzyme digest is performed after adapter ligation with the original cfDNA fragments. The resulting RRMP library should be able to preserve the length and end of cfDNA. The results of sequence length analysis showed that the RRMP datasets retained the length characteristics of cfDNA with a distribution peak of around 170 bp (Additional file 2: Fig S6 e), suggesting that RRMP has the potential to allow the simultaneous analysis of cfDNA methylation, fragmentation, and end motifs.

Next, we investigated the predictive potential of RRMP data for breast cancer detection. Six samples were excluded due to insufficient coverage. 1547 CpGs in the promoter with coverage > 100 × in most samples were used to develop BC detection model using a supervised machine learning approach with LOO cross-validation (Additional file 1: Table S13). Within each fold, 49 samples were used to identify differentially methylated CpGs and to train the model to distinguish BC from NBC, and the remaining one sample was used to evaluate the performance of each model. The predicted results of all samples were used to calculate the AUC to represent the discrimination performance of BC and NBC and the result was 0.85 (Fig. 4e, f).

## Discussion

Methylation of CpG dinucleotides is an important epigenetic modification and plays a crucial role in cell differentiation, proliferation, transcriptional regulation, and maintenance of genomic stability [31]. Global methylation analysis can be achieved by methods such as WGBS and RRBS; however, cost-effectively analyzing low input and highly fragmented DNA remains challenging. In this study, we introduce RRMP, which uses the opposite digestion strategy of RRBS to achieve efficient enrichment and deep sequencing of CG-rich sequences and can analyze the specific methylation regions of tumor cells.

Compared to the traditional RRBS, the RRMP has obvious advantages, as it is a depletion after library preparation. RRMP is compatible with currently used methylation analysis methods such as WGBS, EM-seq, and targeted methylation sequencing by hybridization capture, and it is suitable for limited clinical samples or highly fragmented cfDNA samples [16, 17, 32]. The RRMP library preserves the length and end of the original fragments. Thus, tumor-related information such as methylation signal, fragmentation, and end motifs can be analyzed simultaneously.

The methylation signal of circulating tumor DNA can be used as a liquid biopsy marker for tumor detection. Global methylation analysis of cfDNA is helpful for discovery of efficient tumor methylation markers [12, 25]. We analyzed plasma samples from breast cancer and non-breast-cancer controls by RRMP and obtained methylation sequencing data enriched in CpG islands and promoters. Based on the CpG methylation signals in the promoter regions, the predictive model was able to distinguish breast cancer patients from controls, including patients with breast nodules and mastitis.

Although we developed a new method for whole gene methylation sequencing, there were also several limitations in this study. First, our approach was tested on a relatively low number of patients. Validation in a larger cohort can further demonstrate the reliability of the RRMP data for tumor detection. Second, although RRMP can enable genome-wide methylation analysis and has the potential to detect all tumor-associated methylation, the ability of RRMP to detect tumors other than breast cancer and to trace tumor tissue needs to be validated in clinical samples. Finally, we did not further analyze fragmentation and end motif of cfDNA using RRMP data to achieve better differentiation of breast cancer. Integrating multi-modal features from RRMP to enhance cancer detection is an important goal of our future work.

### Supplementary Information

**Additional file 1**: **Fig. S1**. In silico restriction digestion analysis.** a** The amount of remaining reads from AT-rich reads deletion of a public WGBS dataset for K562 cells.** b** The CG rate of remaining reads from AT-rich reads deletion of a public WGBS dataset for K562 cells, compared with a public RRBS dataset for K562 cells.** c** The percentage of remaining reads mapping to CpG island, CpG shore or CpG shelf, compared to the original WGBS dataset and a RRBS dataset.** d** The percentage of remaining reads mapping to promoter, compared to the original WGBS dataset and a RRBS dataset. **Fig. S2**. Genome plot for the GBGT1 gene locus compares read coverage between and WGBS, XRBS, RRBS, 1-cut RRMP and 4-cut RRMP. Boxes represent reads, and unmethylated (blue) and methylated (red) CpGs are indicated. CpG islands are indicated. **Fig. S3**. RRMP efficiently captures CpGs in CpG islands and promoters. a, b, Plots show the number of CpG islands (**a**) or promoters (**b**) with at least 100-fold combined coverage as a function of sequencing depth (x axis) for 4-cut RRMP(K562), XRBS (K562), WGBS (K562) and RRBS (SW1353). Enrichment for functional elements at a uniform sequencing depth of 10 billion base pairs is indicated. Vertical gray line indicates break in x-axis scale.** c** Plot compares CpG coverage as a function of sequencing depth (x-axis) for WGBS, XRBS, RRBS, 1-cut RRMP and 4-cut RRMP. **d**,** e**, Downsampling analysis plot as in panel c but restricted to CpGs within CpG islands (**d**) and gene promoters (**e**).** f** Heat map shows genome-wide DNA methylation in 100-kb windows for 1- cut RRMP, XRBS, WGBS from K562 cells. **Fig. S4**. RRMP detects tumor-related methylation differences.** a** Heat map shows Pearson correlation of RRMP methylation profiles of 100 kb windows generated from 9 tumor cell lines and 3 WBC samples from healthy donors.** b** Heat map depicts hypermethylated and hypomethylated regions in each type of tumor cells compared to WBC samples. **Fig. S5**. Cell-line-specific DNA hypomethylation from RRMP correlates with H3K27ac signal. Heat map depicts 8-kb regions centered on H3K27ac peaks identified in NCI-H460 and HT29 ChIP-seq datasets. Rows are ordered by DNA methylation difference between both cell lines. Peaks not specifically hypermethylated in either cell line (‘Others’) were downsampled for visualization. **Fig. S6**. RRMP efficiently captures CpGs in CpG islands and promoters using cfDNA.** a** Plot compares CpG coverage as a function of sequencing depth (x-axis) for 4-cut RRMP and EM-seq. **b**,** c**, Downsampling analysis plot as in panel c but restricted to CpGs within CpG islands (b) and gene promoters (**c**).** d** Plot shows coverage depth of CpGs in 4-cut RRMP and EM-seq at a uniform sequencing depth of 10 billion base pairs.** e** Length distribution of cfDNA fragments from breast cancer patients (BC, n = 29) and non-breast cancer individuals (NBC, n = 27).

**Additional file 2**: **Supplementary Table 1**. Quality control metrics for all datastes in this study. **Supplementary Table 2**. DMRs in liver cancer cell lines comparing to WBCs. **Supplementary Table 3**. DMRs in lung cancer cell lines comparing to WBCs. **Supplementary Table 4.** DMRs in gastric cancer cell lines comparing to WBCs. **Supplementary Table 5.** DMRs in colorectal cancer cell lines comparing to WBCs. **Supplementary Table 6.** DMRs in breast cancer cell lines comparing to WBCs. **Supplementary Table 7.** Hypermethylated regions in five types of cancer cell lines comparing to WBCs. **Supplementary Table 8**. Hypomethylated regions in five types of cancer cell lines comparing to WBCs. **Supplementary Table 9**. Hypermethylated regions in each type of cancer cell lines comparing to WBCs. **Supplementary Table 10.** Hypomethylated regions in each type of cancer cell lines comparing to WBCs. **Supplementary Table 11**. Characteristics of patients for RRMP analysis. **Supplementary Table 12**. 1547 CpGs in promoters for BC detection. **Supplementary Table 13.** Public datasets utilized in this study.

## Data Availability

The raw sequence data reported in this paper have been deposited in the Genome Sequence Archive [33] in National Genomics Data Center [34], China National Center for Bioinformation/Beijing Institute of Genomics, Chinese Academy of Sciences (GSA-Human: HRA004992) that are publicly accessible at https://ngdc.cncb.ac.cn/gsa-human. A full list of the public datasets used in this study is provided in Additional file 1: Table S2.

## Abbreviations

RRMP: Reduced representative methylome profiling
cfDNA: Cell-free DNA
ctDNA: Circulating tumor DNA
WGBS: Whole-genome bisulfite sequencing
RRBS: Reduced representative bisulfite sequencing
XRBS: Extended-representation bisulfite sequencing
WBC: Whole blood cell
LOO: Leave one out
DMR: Differentially methylated region
BC: Breast cancer patients
NBC: Non-breast cancer individuals
ROC: Receiver operator characteristic curves
AUC: Area under the curve
