# Supplementary material for: Reduced representative methylome profiling of cell-free DNA for breast cancer detection
Source: Clin Epigenetics. 2024 Feb 27;16:33. doi: 10.1186/s13148-024-01641-x (PMC10898043; doi:10.1186/s13148-024-01641-x)
Supplement: Supplementary file 2 — Additional file 2: Supplementary Table 1. Quality control metrics for all datastes in this study. Supplementary Table 2. DMRs in liver cancer cell lines comparing to WBCs. Supplementary Table 3. DMRs in lung cancer cell lines comparing to WBCs. Supplementary Table 4. DMRs in gastric cancer cell lines comparing to WBCs. Supplementary Table 5. DMRs in colorectal cancer cell lines comparing to WBCs. Supplementary Table 6. DMRs in breast cancer cell lines comparing to WBCs. Supplementary Table 7. Hypermethylated regions in five types of cancer cell lines comparing to WBCs. Supplementary Table 8. Hypomethylated regions in five types of cancer cell lines comparing to WBCs. Supplementary Table 9. Hypermethylated regions in each type of cancer cell lines comparing to WBCs. Supplementary Table 10. Hypomethylated regions in each type of cancer cell lines comparing to WBCs. Supplementary Table 11. Characteristics of patients for RRMP analysis. Supplementary Table 12. 1547 CpGs in promoters for BC detection. Supplementary Table 13. Public datasets utilized in this study. [file 13148_2024_1641_MOESM2_ESM.pdf]

Figure S1

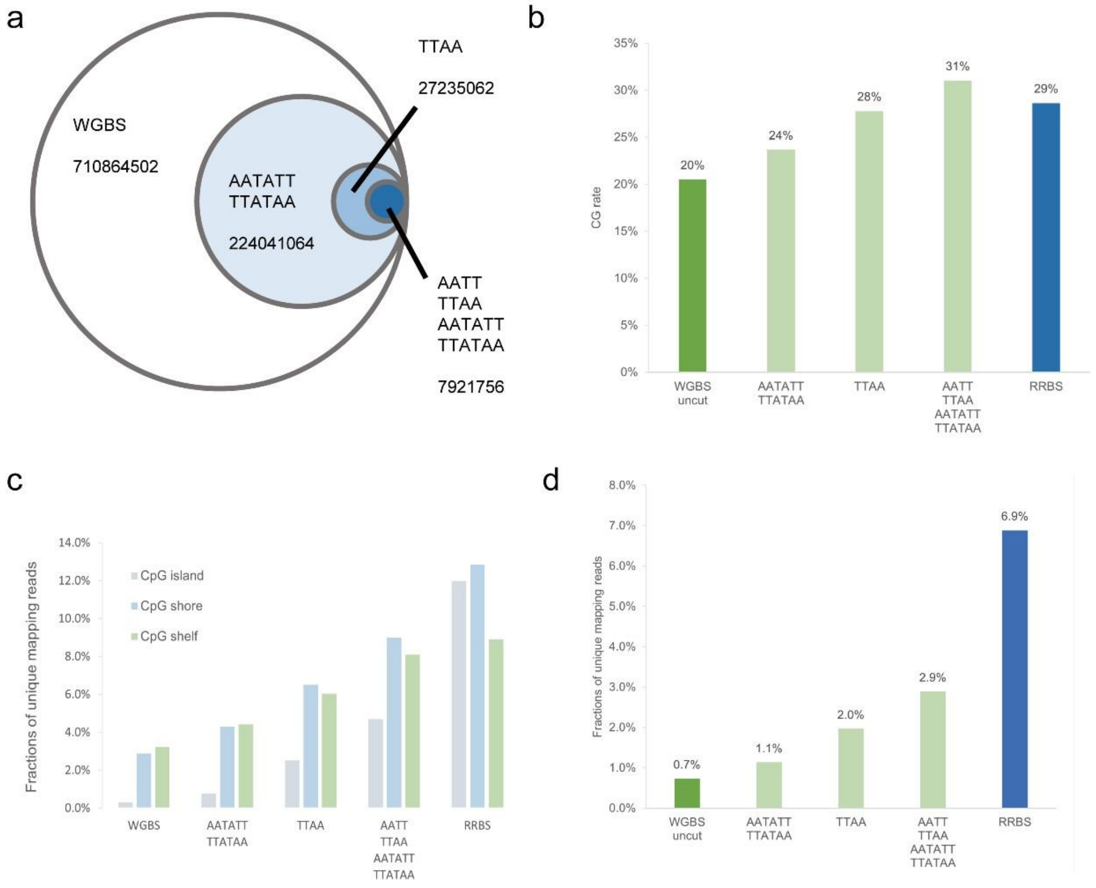

Fig. S1| In silico restriction digestion analysis. a, The amount of remaining reads from AT-rich reads deletion of a public WGBS dataset for K562 cells. b, The CG rate of remaining reads from AT-rich reads deletion of a public WGBS dataset for K562 cells, compared with a public RRBS dataset for K562 cells. c, The percentage of remaining reads mapping to CpG island, CpG shore or CpG shelf, compared to the original WGBS dataset and a RRBS dataset. d, The percentage of remaining reads mapping to promoter, compared to the original WGBS dataset and a RRBS dataset.

Figure S2

a

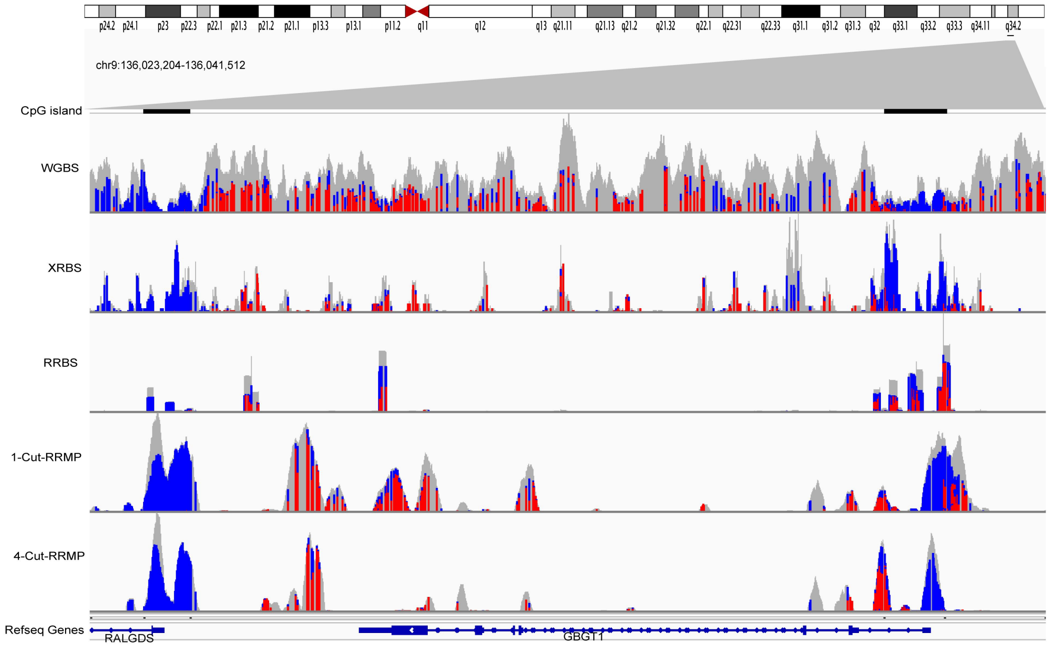

Fig. S2 | Genome plot for the GBGT1 gene locus compares read coverage between and WGBS, XRBS, RRBS, 1-cut RRMP and 4-cut RRMP. Boxes represent reads, and unmethylated (blue) and methylated (red) CpGs are indicated. CpG islands are indicated.

Figure S3

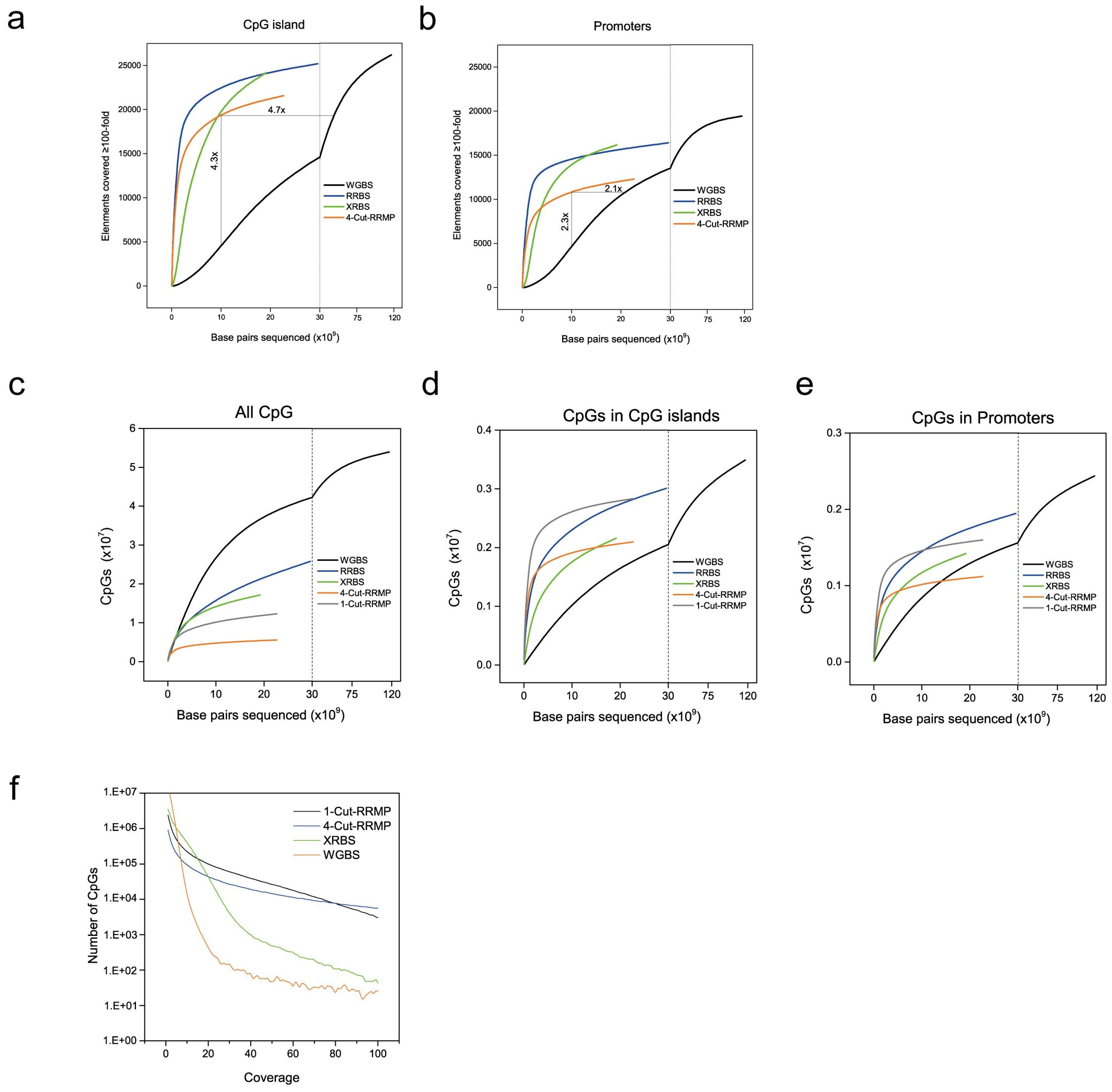

Fig. S3 | RRMP efficiently captures CpGs in CpG islands and promoters. **a, b,** Plots show the number of CpG islands (**a**) or promoters (**b**) with at least 100-fold combined coverage as a function of sequencing depth (x axis) for 4-cut RRMP(K562), XRBS (K562), WGBS (K562) and RRBS (SW1353). Enrichment for functional elements at a uniform sequencing depth of 10 billion base pairs is indicated. Vertical gray line indicates break in x-axis scale. **c**, Plot compares CpG coverage as a function of sequencing depth (x-axis) for WGBS, XRBS, RRBS, 1-cut RRMP and 4-cut RRMP. **d,e,** Downsampling analysis plot as in panel **c** but restricted to CpGs within CpG islands (**d**) and gene promoters (**e**). **f,** Heat map shows genome-wide DNA methylation in 100-kb windows for 1-cut RRMP, XRBS, WGBS from K562 cells.

Figure S4

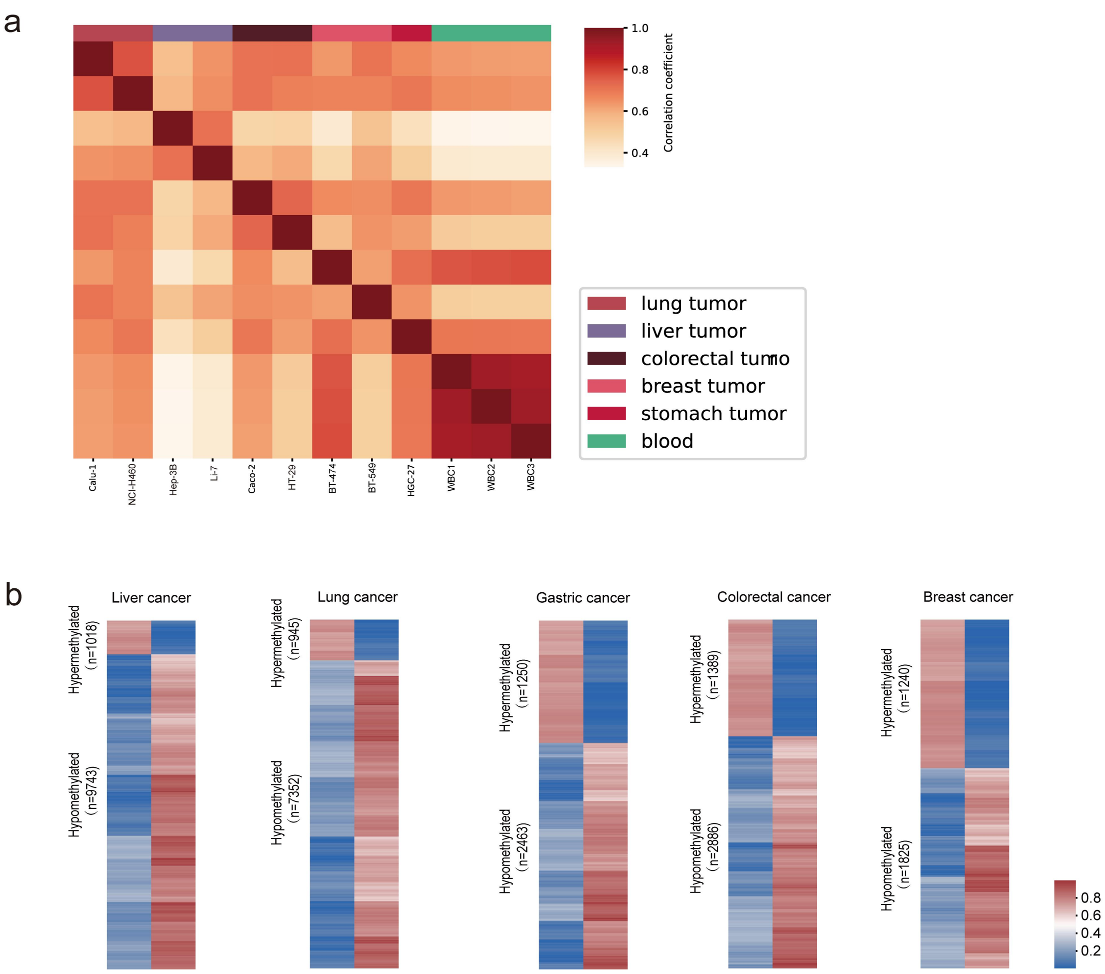

Fig. S4 | RRMP detects tumor-related methylation differences. **a,** Heat map shows Pearson correlation of RRMP methylation profiles of 100kb windows generated from 9 tumor cell lines and 3 WBC samples from healthy donors. **b,** Heat map depicts hypermethylated and hypomethylated regions in each type of tumor cells compared to WBC samples.

Figure S5

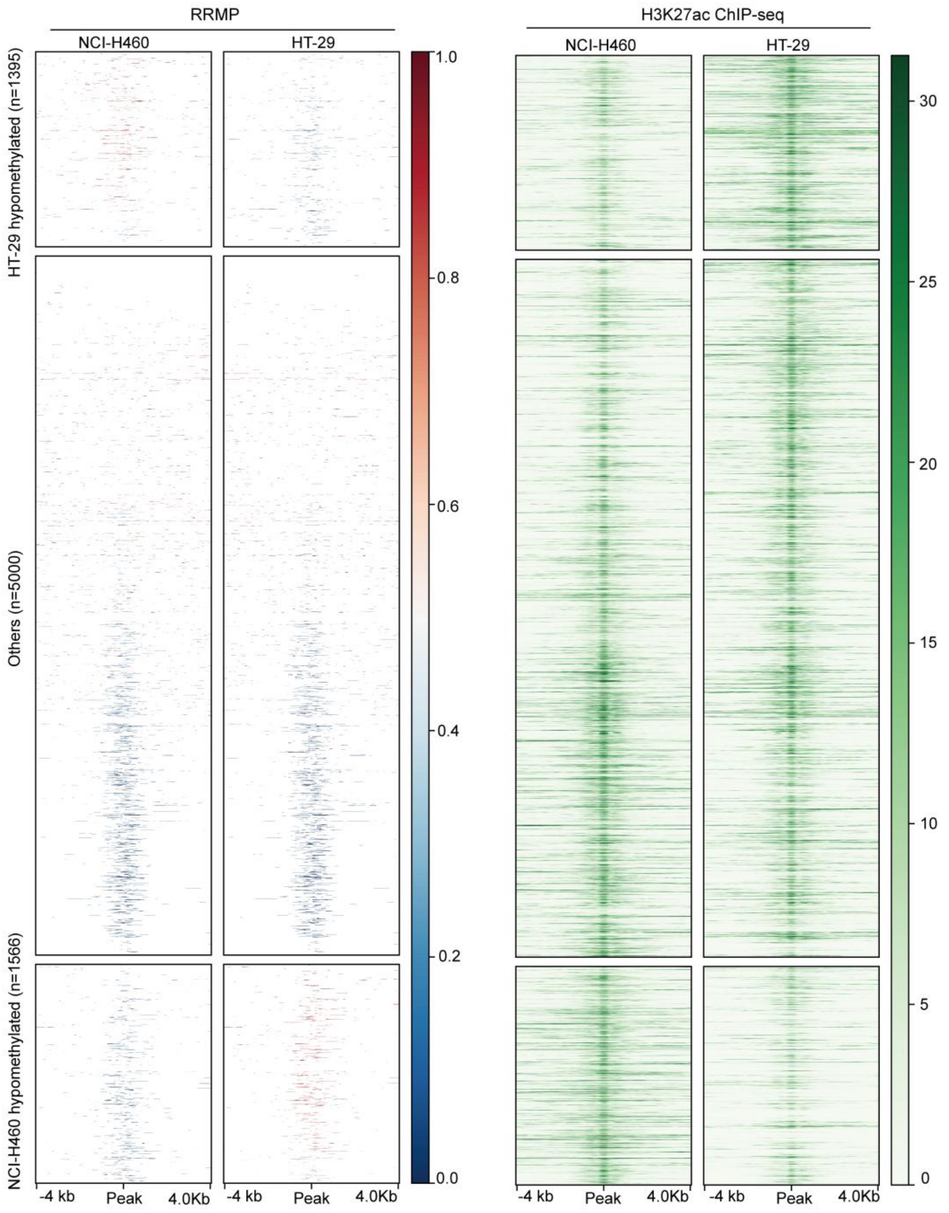

Fig. S5 | Cell-line-specific DNA hypomethylation from RRMP correlates with H3K27ac signal. Heat map depicts 8-kb regions centered on H3K27ac peaks identified in NCI-H460 and HT29 ChIP-seq datasets. Rows are ordered by DNA methylation difference between both cell lines. Peaks not specifically hypermethylated in either cell line ('Others') were downsampled for visualization.

Figure S6

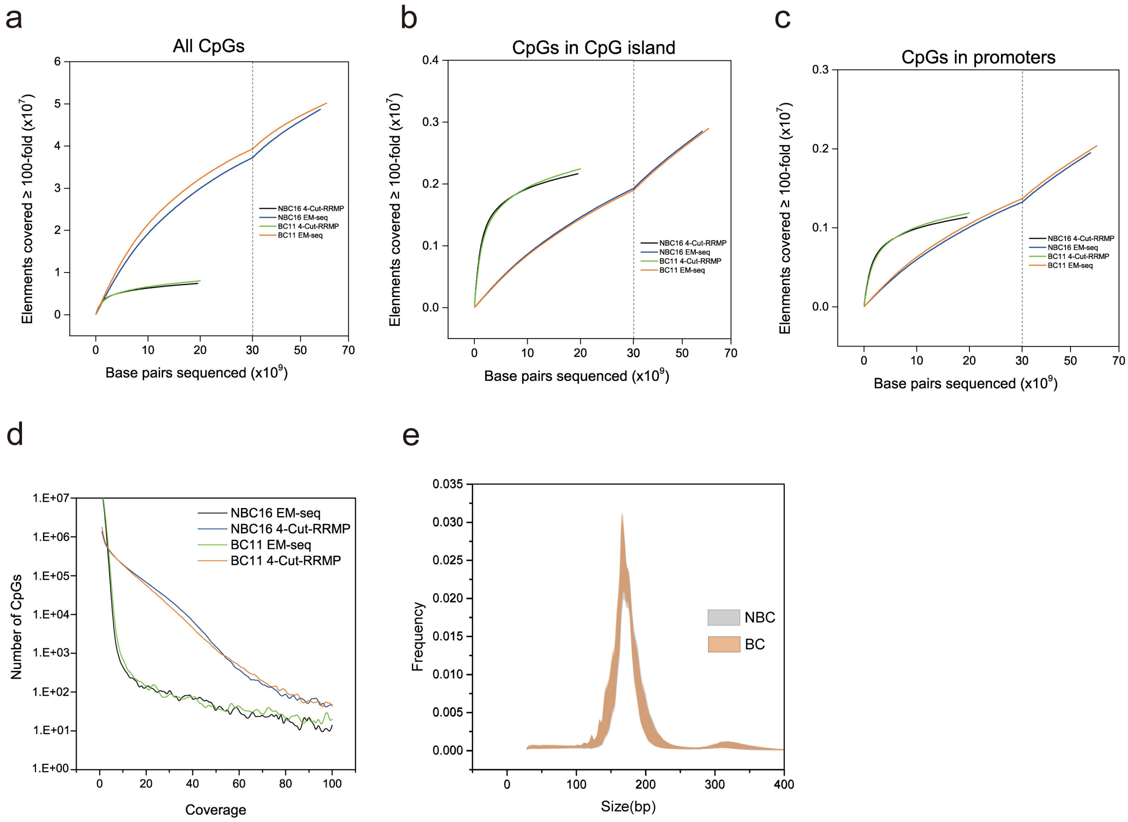

Fig. S6 | RRMP efficiently captures CpGs in CpG islands and promoters using cfDNA. **a,** Plot compares CpG coverage as a function of sequencing depth (x-axis) for 4-cut RRMP and EM-seq. **b, c,** Downsampling analysis plot as in panel c but restricted to CpGs within CpG islands (**b**) and gene promoters (**c**). **d,** Plot shows coverage depth of CpGs in 4-cut RRMP and EM-seq at a uniform sequencing depth of 10 billion base pairs. **e,** Length distribution of cfDNA fragments from breast cancer patients (BC, n=29) and non-breast cancer individuals (NBC, n=27).
